# Assessing the Knowledge and Attitude toward COVID-19 Vaccination in Saudi Arabia

**DOI:** 10.3390/ijerph18158185

**Published:** 2021-08-02

**Authors:** Heba M. Zahid, May A. Alsayb

**Affiliations:** Department of Medical Laboratories Technology, College of Applied Medical Sciences, Taibah University, P.O. Box 344, Madinah 42353, Saudi Arabia; msayb@taibahu.edu.sa

**Keywords:** COVID-19, vaccine hesitancy, vaccine acceptance, vaccine attitude, Saudi Arabia

## Abstract

COVID-19 was declared a pandemic by the WHO in March 2020. The most promising strategy to control the pandemic was to develop a vaccine. However, vaccination hesitancy is a major threat to world public health. Understanding the reasons behind this hesitancy might help in developing encouragement strategies. This cross-sectional study aimed to assess the knowledge and attitude toward the COVID-19 vaccine in Saudi Arabia. A total of 1599 responses were received; the overall vaccine acceptancy was 79.2%. Age, sex, and nationality of participants significantly predicted the vaccination status. A significantly higher proportion of participants, who reported being vaccinated, or intended to receive the vaccine, stated that the COVID-19 infection is dangerous, or varies from person to person; the vaccine is safe, and think there is a definite need for the vaccine (*p* < 0.001). The major encouragement factors to receive the vaccine were either confidence in the government decisions (54.8%), or the feeling of responsibility to stop the pandemic (48.7%), whereas the main discouraging factors were concerns about the insufficient clinical trials (11.4%), or the undiscovered side effects (11%). The results of this study indicate good acceptance toward the COVID-19 vaccine among residents of Saudi Arabia.

## 1. Introduction

The severe acute respiratory syndrome coronavirus 2 (SARS-CoV-2) is the cause of the pandemic upper respiratory infection coronavirus disease 2019 (COVID-19). Due to the SARS-CoV-2’s highly transmissible and complicated pathological outcome, the COVID-19 infection is a major threat globally, [1]. not only for public health, but also economically. According to the global COVID-19 dashboard by Johns Hopkins university, the total global number of COVID-19 cases to date is 173,766,202, with 3,740,609 deaths worldwide [2]. Since the spread of this infection in Saudi Arabia, 459,968 positive cases were reported with 7488 deaths [3]. Although the elderly and patients with chronic diseases are among these mortalities, the severity of the disease is not predictable as some healthy young individuals lost their lives as well. The treatment strategies are not well defined due to the conflicting results of the ongoing trials. These data indicate indeed the unpredictable severity of this infection and the need for urgent resolution to stop the pandemic. The availability of vaccines is the most promising strategy for ending the crisis.

COVID-19 global crises unified the science community to understand the pathological mechanism and immune response of SARS-CoV-2. After a year of collaborative work, some vaccines succeeded and passed phase 3 clinical trials [4]. In late 2020, COVID-19 vaccines were approved, and by early 2021 vaccine companies started exporting the doses to different countries. Vaccines developed by PfizerBioNTech and AstraZeneca were approved in Saudi Arabia and an online vaccination booking system was developed to prioritize the vaccination to the elderly, patients with chronic diseases and medical staff. The government has offered the vaccine for all Saudi and non-Saudi residents. Yet, vaccine hesitancy is a public controversial topic that can affect the government immunization plan to stop the pandemic.

Several studies have highlighted that COVID-19 vaccine hesitancy among the public could be a potential threat to global public health [5,6,7,8,9]. However, little is known about the reasons and factors behind vaccine hesitancy in the Saudi population. The reasons for this hesitancy could vary from misinformation regarding the vaccine efficacy and safety to misconceptions about the need for vaccination. Vaccine hesitancy might be affected by culture and sociodemographic characteristics. Therefore, the current study aims to assess the public awareness of the available vaccines, their attitude toward the vaccination, and the reasons behind this attitude. The resulted data might be important for the government to target the main reasons behind vaccine hesitancy among the public and implement an awareness campaign if needed. Assessing the level of vaccination hesitancy and the reasons behind it will help in developing an encouragement strategy through an awareness campaign and social media influencers.

## 2. Materials and Methods

### 2.1. Study Population

The inclusion criteria included all residents of Saudi Arabia 18 years old and above. 

There were no exclusion criteria specified in this study. The latest Kingdom of Saudi Arabia (KSA) census was used to determine the required sample size to achieve the study objectives and sufficient statistical power [10]. The sample size needed was calculated with a sample size calculator [11]. Using a margin of error of ±4%, a confidence level of 99%, a 50% response distribution, and 34,218,169 people, a sample size of 1037 was needed. Reducing the margin of error to ±2% will increase the sample size to 4147. So, our target was between 1037 and 4147. 

### 2.2. Data Collection

Cross-sectional data were collected between 18 March and 7 April 2021 via online survey, Google Forms. The online surveying method was used to maximize reach and gather data from as many respondents as possible. The questionnaire was distributed conveniently through multiple social media applications (WhatsApp and Twitter) that are commonly used in Saudi Arabia. The questionnaire was available in both languages, English and Arabic. The online survey was distributed to family members, friends, staff and students of many faculties in different colleges and universities (male and female campuses) around Saudi Arabia. Additionally, we asked these potential participants to help in the survey distribution. A number of social media activists were also contacted to help in distributing the survey. To ensure the representativeness of the sample included in the study, and to maximize the generalizability of the study findings, participants from disadvantaged communities were also targeted. Several individuals who were using the services of welfare society centers in multiple cities in Saudi Arabia were contacted. In fact, during data collection, a proportion of participants, based on sex, age group, income, and education level were monitored, and a smaller proportion of male participants were observed. Thus, males were specifically targeted on social media to ensure balanced data, but responses from male participants were still low.

Data collected via the survey included sociodemographics (region of residency, age, sex, marital status, education level, employment status, family income per month). Data concerning the vaccination status (vaccinated, intending to receive the vaccine, not intending to receive the vaccine) and reasons explaining the vaccination status were also collected.

### 2.3. Statistical Analysis

Data were analyzed by the Statistical Package for Social Sciences (IBM^®^ SPSS^®^ Statistics for Windows, Version 27.0. Armonk, NY, USA: IBM Corp). Descriptive data were presented as frequencies and percentages. The chi-square test was used to assess the relationships between two categorical variables. Logistic regression analysis was performed to investigate predictors associated with vaccination status. Potential predictors were coded as follows: Region of residency (western = 1, central = 2, other = 3); sex (male = 1, female = 2); age group (18–30 years = 1, 31–40 years = 2, 41–50 years = 3, >50 years = 4); nationality (Saudi = 1, non-Saudi = 2); marital status (single = 1, married = 2); education level (≤high-school = 1, university/postgraduate = 2); employment status (unemployed = 1, student or employee in the health field = 2, student or employee in non-related health field = 3); family income per month (<SR 4000 = 1, SR 4000–1000 = 2, ≥SR 10000 = 3). The outcome was the vaccination status (vaccinated or intended to receive the vaccine = 1, not intending to receive the vaccine = 2). A *p*-value of <0.05 was considered statistically significant.

### 2.4. Ethical Consideration

This study was approved by the scientific research and ethics committee at the College of Applied Medical Sciences, Taibah University, Madinah (2021/91/115/MLT). The first page of the questionnaire stated the aim and objectives of the study and contained the agreement of participation for individuals > 18 years.

## 3. Results

The total number of participants included in this study was 1599, where 68.8% (*n* = 1100) were recruited from the western region of Saudi Arabia. Three-quarters (75.8%) of the participants were females, while 90.5% (*n* = 1447) of the sample were Saudis. Two-thirds of the sample were married (*n* = 1081), and over half of the participants (57.3%, *n* = 916) reported a family income of >SR 10,000 a month. The number of participants who reported being vaccinated was 590 (36.9%), whereas the number of participants who intended to be vaccinated was 677 (67.1%) from all participants who are unvaccinated at the time the data for this study was collected.

### 3.1. Sociodemographic Characteristics of Participants

Sociodemographic characteristics of participants included in this study were similar across the different groups of vaccination, except that sex and nationality were statistically different. The percentage of participants who are vaccinated or intended to receive the vaccine was 84.3% (*n* = 167) of males and 76.5% (*n* = 475) of females (*p* = 0.019). Participants who are vaccinated or intended to receive the vaccine were 80.2% (1161) Saudi and 69.7% (*n* = 106) non-Saudi (*p* = 0.002). Detailed descriptions of the sociodemographic characteristics of participants stratified by vaccination status are provided in Table 1.

### 3.2. Knowledge Related to COVID-19 Infection and Vaccine

Data concerning knowledge related to the COVID-19 infection and vaccine showed that 2.30% (*n* = 36) of participants think that the COVID-19 infection is not dangerous. About half of the sample (*n* = 868) think the COVID-19 vaccines are safe, while 76.0% (*n* = 1215) of participants think there is a definite need for the vaccine. A significantly higher proportion of participants who reported being vaccinated, or intended to receive the vaccine, stated that COVID-19 infection is dangerous or varies from person to person, that the vaccine is safe, and think there is a definite need for the vaccine (*p* < 0.001, for all). Sources of information related to COVID-19 infection and vaccine that were statistically significantly different among the vaccination groups included: doctors and other healthcare professionals (85.4%, *n* = 580); awareness campaigns (91.3%, *n* = 597); family and friends (86.2%, *n* = 313); other websites (56.6%, *n* = 47), (*p* < 0.05, for all). All data concerning knowledge related to COVID-19 infection and sources of information in relation to vaccination status are described in Table 2.

Stratification by sex was performed, revealing no significant difference between males and females regarding how dangerous COVID-19 infection is, if participants think the vaccine is safe, and the participants’ understanding of the need for a vaccine. Regarding the sources of information, we found differences in the association among males and females. A significantly higher proportion of female participants, who are not intending to take the vaccine, did not use doctors and other healthcare professionals as sources of information, compared to female participants who used doctors and other healthcare professional as sources of information (29.3% vs. 14.2%, respectively, *p* < 0.001), while no significant differences were found among males in both groups (13.3% vs. 18.3%, *p* = 0.339). Associations between the awareness campaign, use of social media, and use of medical websites as sources of information and vaccination status were similar among males and females (*p* < 0.05). A significantly higher proportion of female participants who were not intending to take the vaccine did not use friends and family as sources of information, compared to female participants who used friends and family as sources of information (26.3% vs. 15.0%, respectively, *p* = 0.004), while no significant differences were found among males in both groups (14.9% vs. 18.2%, *p* = 0.601). No significant differences were found among females who did not use and who used other websites as a source of information (22.8% vs. 36.7%, *p* = 0.082). Among males, a higher proportion of participants who used other websites as a source of information were not intending to take the vaccine in comparison to participants who did not use other websites as a source of information (43.8% vs. 13.2%, respectively, *p* = 0.001).

Logistic regression analysis shows that participants’ sex predicted vaccination status, where female participants had higher odds of not being vaccinated, nor intending to receive the vaccine, compared to males (odds ratio (OR) = 1.66 (95% confidence interval (CI): 1.08–2.54), *p* = 0.020). The age of participants also predicted the vaccination status, where participants aged >50 years had higher odds of not being vaccinated, nor intending to receive the vaccine (OR = 1.51 (95% CI: 1.02–2.22), *p* = 0.038), compared to participants aged between 18–30 years. The data show also that Saudis had lower odds for not being vaccinated, nor intending to receive the vaccine (OR = 0.57 (95% CI: 0.39–0.82), *p* = 0.003), compared to non-Saudi participants. All other sociodemographic characteristics did not predict the vaccination status.

### 3.3. Attitudes toward COVID-19 Vaccination

Multiple factors affected the vaccination decision. For instance, respondents who were vaccinated, or intended to be vaccinated were encouraged mainly because of their confidence in the government’s decisions during the pandemic (54.8%), they felt it is their duty to eliminate the pandemic (48.7%), or they were scared of the infection and wanted to travel abroad (31.2% and 31.6%, respectively). The death of a relative with the disease was not a major factor in encouraging the respondents to be vaccinated (6.6%). On the other hand, the main reasons for vaccine hesitancy were either the lack of confidence in the adequacy of clinical trials related to the vaccine, or concerns about the side effects (11.4% and 11%, respectively). All data concerning reasons explaining attitudes toward COVID-19 vaccination are described in Table 3.

## 4. Discussion

In this study we assessed the knowledge and attitude of 1599 participants toward COVID-19 vaccine across all regions in Saudi Arabia covering various ages. Of these, 36.9% of participants are vaccinated, 42.3% have the intention to be vaccinated, and 20.8% do not intend to be vaccinated. The Saudi population total acceptance (vaccinated and intending to be vaccinated) in our study was 79.2%, which is higher than what has been reported in another study that demonstrated 64.72% acceptance intention to COVID-19 [10]. Although the finding of this previous work was carried out before vaccine approval, the increase in the acceptance level could reflect an increase in the general public awareness level. The Saudi population acceptance toward COVID-19 vaccination in this study is 79.2%, which is considered high compared to other Middle Eastern countries, such as Kuwait (53.1%) and Jordan (37.4%) [8,12]. The vaccination hesitancy among the general population has been studied worldwide in different countries. Acceptance rate varied among countries, and among studies within the same country [6]. This indicates that the general populations’ attitude toward the vaccine is continuously changing over time. Several explanations could play a role in such variations; for instance, the variety of vaccine companies, the emergence of different mutations within the SARS-CoV-2 virus, the increase in the reported incidence number, and the spreading of incorrect information from unauthorized parties.

The variation in sociodemographic characteristics affect vaccine hesitancy globally [7]. The current study showed that male acceptance toward the COVID-19 vaccine was statistically higher than the female participants (*p* = 0.019). This is also observed in other studies [8,9], possibly due to job requirements and other social obligations. Moreover, the acceptance of Saudi participants toward the COVID-19 vaccine was statistically higher than non-Saudi participants (*p* = 0.002). This could be attributed to the low number of non-Saudi participants in the current study (*n* = 152). There was no association between age and vaccination status; however, in a logistic regression model, age was a predictor for vaccination acceptancy, as younger people were more likely to be vaccinated than older people. This could possibly be due to social activities that necessitated vaccination for participation.

The majority of participants were aware of the severity variation of the COVID-19 infection. The KSA government has adapted an approach to keep the public updated, with daily reports regarding the number and severity of COVID-19 cases. This continuous effort to increase public awareness toward the severity of COVID-19 has indeed increased the awareness level in the majority of our participants.

Vaccine effectiveness is one of the reasons for vaccine hesitancy among the population. A previous study has reported that 49.9% of their participants agreed that the effectiveness of the vaccine is the reason for their hesitancy [13]. Similarly, in our study, the majority of participants agreed that there is a need for vaccination; yet, safety and efficacy of the vaccine has been the major cause of hesitancy in the general population. Fifty four percent of participants think the vaccine is safe, while still 36.6% admitted they do not know. Twenty percent of participants do not intend to be vaccinated, and most of their responses were related to side effects, and insufficient clinical studies. The controversy of the AstraZeneca vaccine safety was a hot topic in the science community recently. The company announced 79% efficacy of the COVID-19 vaccine but, later, the result of clinical trial III was released, and the company was accused of misrepresenting the results. Accordingly, on 25 March 2021, they declared an efficacy of 76%, based on key findings from the clinical trials III. These controversial changes in the results caused public hesitancy. However, it is worth noticing that the difference in the efficacy is still close and considered a good result [14]. Furthermore, the side effects that have been reported with the vaccines could have affected public hesitance toward the vaccines. Several cases were reported of thrombosis with thrombocytopenia and, in some cases, bleeding, in people who received AstraZeneca vaccine [15,16,17,18,19,20]. These reports were reviewed by the European medicines agency (EMA), and they have announced these side effects are considered rare, and confirmed the positivity of the overall benefits of the AstraZeneca vaccine; while also highlighting the importance of declaring these side effects to the public [21,22,23]. 

We analyzed the reasons behind participants’ thoughts toward the COVID-19 vaccination, and observed high heterogeneity in the responses explaining attitudes toward COVID-19 vaccination. Different factors can play a role in influencing participants’ decisions. According to our results, confidence in the decisions of the government of the KSA was the highest factor influencing participants positively toward vaccination. This could be the result of the risk management plan and the precautionary measures that the KSA has followed during the COVID-19 pandemic. The public trust in the government has also been shown to enhance the acceptance of COVID-19 vaccination in countries, such as the Asian nations [7]. The Global Entrepreneurship Monitor 2020/2021 report indeed reported that the Saudi Arabian government had the highest score worldwide in response to the COVID-19 pandemic. Saudi Arabia did not only succeed in the governmental response but also was the highest in entrepreneurship response among worldwide economies that focused on their ability to introduce new approaches for business opportunities and services that were adapted to the situations, and even new cooperation nationally and internationally [24]. The continued effort of KSA during the pandemic was obvious to the public, and gained the confidence and the trust of 54.8% of the participants. The second highest response was “My duty to the community to participate in the eradication of the pandemic.” This reflects the high ethics of individuals toward the community and the high trust in the health system; the higher the trust in health system the more acceptance toward vaccination [25].

Utilizing the COVID-19 health campaign to educate the community scientifically is of high importance to increase health awareness and ensure its sustainability. Our study has identified that the source of information between the vaccine accepting group and non-accepting group was statistically different. The awareness of the COVID-19 vaccine campaign should focus on the general public’s concerns and misconception, and should utilize these factors, not only to announce the safety and effectiveness of the vaccine, but also provide trusted sources of information that increase the public’s general knowledge and create a well-educated community.

## 5. Conclusions

The overall response of participants in this study indicates good acceptance toward the COVID-19 vaccine among individuals. A total of 79.2% of participants were vaccinated or intending to be vaccinated by the time of data collection. The majority of the participants knew that the COVID-19 infection varies from person to person, that the vaccine is safe, and there is a definitive need for vaccination. However, these results are influenced by the vaccinated, or intended to be vaccinated group. The confidence in the decisions of the government and the feeling of responsibility toward the community were the main reasons that encouraged the respondents to be vaccinated. However, the belief of insufficient clinical trials, and not well-identified side effects were the main reasons for vaccination hesitancy. Age, gender, and nationality were strong predictors for vaccination acceptancy. The use of the online questionnaire limited the access to various groups. The majority of participants were from the western region; hence, other regions’ presentation was very low. The non-Saudi participation was also low, which might be due to the unsuitability of the questionnaire language for some nationalities.

## Figures and Tables

**Table 1:** Sociodemographic characteristics of participants stratified by vaccination status.

	Vaccinated or Intended to Receive the Vaccine (*n* = 1267)	Not Intended to Receive the Vaccine (*n* = 332)	Total (*n* = 1599)	*p*
**Region of residency**				
Western	876 (79.6)	224 (20.4)	1100 (68.8)	0.388
Central	225 (80.4)	55 (19.6)	280 (17.5)	
Other	166 (75.8)	53 (24.2)	219 (13.7)	
**Sex ^1^**				
Female	475 (76.5)	146 (23.5)	621 (75.8)	0.019 *
Male	167 (84.3)	31 (15.7)	198 (24.2)	
**Age Group**				
18–30 years	418 (76.4)	129 (23.6)	547 (34.2)	0.153
31–40 years	415 (79.5)	107 (20.5)	522 (32.6)	
41–50 years	229 (80.9)	54 (19.1)	283 (17.7)	
>50 years	205 (83.0)	42 (17.0)	247 (15.4)	
**Nationality**				
Saudi	1161 (80.2)	286 (19.8)	1447 (90.5)	0.002 *
Non-Saudi	106 (69.7)	46 (30.3)	152 (9.50)	
**Marital Status**				
Single	419 (80.9)	99 (19.1)	518 (32.4)	0.260
Married	848 (78.4)	233 (21.6)	1081 (67.6)	
**Education Level**				
≤High-school	221 (76.7)	67 (23.3)	288 (18.0)	0.248
University/Postgraduate	1046 (79.8)	265 (20.2)	1311 (82.0)	
**Employment Status**				
Unemployed	400 (77.2)	118 (22.8)	518 (32.4)	0.372
Student or employee in the health field	359 (79.8)	91 (20.2)	450 (28.1)	
Student or employee in non-related health field	508 (80.5)	123 (19.5)	631 (39.5)	
**Family Income per Month**				
<SR 4000	130 (74.3)	45 (25.7)	175 (10.9)	0.217
SR 4000–10,000	403 (79.3)	105 (20.7)	508 (31.8)	
>SR 10,000	734 (80.1)	182 (19.9)	916 (57.3)	

^1^ Data regarding sex were collected from 819 participants. * A *p* value < 0.05 was considered statistically significant across groups.

**Table 2:** Knowledge related to COVID-19 infection and vaccine and sources of information stratified by vaccination status.

		Vaccinated or Intended to Get the Vaccine (*n* = 1267)	Not Intended to Get the Vaccine (*n* = 332)	Total (*n* = 1599)	*p*
How dangerous is COVID-19 infection?					
Not dangerous		15 (41.7)	21 (58.3)	36 (2.30)	<0.001 *
Dangerous		319 (86.4)	50 (13.6)	369 (23.1)	
Varies from person to person		913 (78.3)	253 (21.7)	1166 (72.9)	
I don’t know		20 (71.4)	8 (28.6)	28 (1.80)	
Do you think the vaccine is safe?					
Yes		838 (96.5)	30 (3.50)	868 (54.3)	<0.001 *
No		26 (17.9)	119 (82.1)	145 (9.10)	
I don’t know		403 (68.8)	183 (31.2)	586 (36.6)	
What is your understanding of the need for a vaccine?					
Definitely there is a need for a vaccine		1101 (90.6)	114 (9.40)	1215 (76.0)	<0.001 *
No need for a vaccine		19 (15.8)	101 (84.2)	120 (7.50)	
I don’t know		147 (55.7)	117 (44.3)	264 (16.5)	
What is the source of your information? *					
1	Doctors and other healthcare professionals.	580 (85.4)	99 (14.6)	679 (42.5)	<0.001 *
2	Awareness campaigns.	597 (91.3)	57 (8.70)	654 (40.9)	<0.001 *
3	Social media.	598 (80.2)	248 (19.8)	746 (46.7)	0.394
4	Friends and family.	313 (86.2)	50 (13.8)	363 (22.7)	<0.001 *
5	Medical websites.	392 (78.6)	107 (21.4)	499 (31.2)	0.652
6	Other websites.	47 (56.6)	36 (43.4)	83 (5.20)	<0.001 *

* Multiple answers allowed, not = 100%.

**Table 3:** Reasons explaining attitudes toward COVID-19 vaccination.

For Participants Who Are Vaccinated or Intended to Get Vaccinated			
What drives you to get the vaccine? *		*n*	%
1	Confidence in the decisions of the government of the Kingdom of Saudi Arabia.	876	54.8
2	Fear of infection.	499	31.2
3	The death of a relative after corona infection.	106	6.60
4	My duty to the community to participate in the eradication of the pandemic.	779	48.7
5	To be able to travel and practice vaccination related activities.	505	31.6
6	Encouragement from the family.	310	19.4
7	Other reasons.	87	5.40
For Participants Who Do not Want to Be Vaccinated			
Why you do not want to get vaccinated? *		*n*	%
1	Do not believe in the efficacy of the vaccine.	68	4.30
2	Do not believe in the need for the vaccine.	57	3.60
3	Got infected with COVID-19 previously and do not need to be vaccinated.	38	2.40
4	Do not need the vaccine as I am committed to precautionary measures.	77	4.80
5	I think there is a conspiracy about the disease and the vaccine.	61	3.80
6	Not eligible for vaccination, due to allergy, pregnancy, other.	45	2.80
7	Family opposition.	10	0.60
8	Not comfortable with side effects.	176	11.0
9	Not confident of the adequacy of previous clinical trials to lunch the vaccine.	182	11.4
10	Other reasons.	40	2.50

* Multiple answers allowed, not = 100%.

## Data Availability

The data presented in this study are available on request from the corresponding author. The data are not publicly available due to ethical restriction.
